# Transcriptome analysis provides insights into the responses of sweet potato to sweet potato virus disease (SPVD)

**DOI:** 10.1016/j.virusres.2020.198293

**Published:** 2021-04-02

**Authors:** Ryland Bednarek, Maria David, Segundo Fuentes, Jan Kreuze, Zhangjun Fei

**Affiliations:** a Plant Pathology and Plant-Microbe Biology Section, School of Integrative Plant Science, Cornell University, Ithaca, NY, 14853, USA; b Boyce Thompson Institute for Plant Research, Ithaca, NY, 14853, USA; c Virology Laboratory, Crop and Systems Science Division, International Potato Center (CIP), Lima 12, Peru; d USDA-ARS, Robert W. Holley Center for Agriculture and Health, Ithaca, NY, USA

**Keywords:** Sweet potato, Sweet potato chlorotic stunt virus, Sweet potato feathery mottle virus, Sweet potato virus disease, Transcriptome profiling, Salicylic acid-mediated defense response pathway

## Abstract

Sweet potato (*Ipomoea batatas*) ranks among the most important crops in the world and provides nutritional and economic sustainability for subsistence farmers in sub-Saharan Africa. Its production is mainly constrained by sweet potato virus disease (SPVD) caused by the coinfection of two positive-sense single-stranded RNA viruses, sweet potato chlorotic stunt virus (SPCSV) and sweet potato feathery mottle virus (SPFMV). Current understanding of sweet potato responses to SPCSV and SPFMV at the molecular level remains very limited. In this study, we performed deep sequencing of both messenger RNA (mRNA) and small RNA (sRNA) populations in an SPVD-susceptible cultivar ‘Beauregard’ upon viral infection, to identify biological pathways that contribute to both general and specific host responses to these important viral pathogens. We found that pathways related to stress response and signaling were significantly affected by viral infection. sRNA components of these pathways were predominantly affected in late stages of the coinfection by SPCSV and SPFMV. We identified several novel microRNAs that were responsive to viral infection, some of which were predicted to target nucleotide-binding site leucine-rich repeat (NBS-LRR) disease resistance genes. The downregulation of the salicylic acid-mediated defense response pathway in particular seems to be a result of the viral infection process, and can in part explain the susceptible nature of the ‘Beauregard’ cultivar.

## Highlights

• Transcriptome responses in sweet potato infected with SPCSV and/or SPFMV were studied.
• Numerous genes, miRNAs and phasiRNAs were responsive mainly to the dual infection.
• Salicylic acid-mediated pathways play important roles in antiviral defense responses.

## 1 Introduction

Sweet potato (*Ipomoea batatas*) is one of the most important food crops in the world and provides significant economic and nutritional benefits to both growers and consumers (Loebenstein, 2015). The worldwide production of sweet potato was 92 million tons in 2018 with China being the global leader in its production (53 million tons) (FAOSTAT, 2018; http://www.fao.org/faostat/en/). While sub-Saharan Africa (SSA) countries like Nigeria, Tanzania, Ethiopia and Uganda have significant land area dedicated to sweet potato farming, average sweet potato production per hectare in SSA amounts to only around a third of that in China (Low et al., 2017). For SSA habitants, sweet potato has become a major staple and the primary biofortified food security crop, financially supporting a critical number of farmers in the region. All sweet potato varieties are rich in vitamins and minerals including vitamins C, K, E and B, along with potassium, phosphorus and dietary fiber (Bovell-Benjamin, 2010). The orange-fleshed sweet potato also serves as a rich source of the cancer-fighting antioxidant, β-carotene. β-carotene can be converted by human body into vitamin A, which is an essential nutrient that contributes to strong immune systems, healthy skin and vision (Sommer, 2001). The World Health Organization estimates that around 2% of the global population suffers from vitamin A deficiency, predominantly affecting pregnant women and children. Vitamin A deficiency in SSA however, is estimated to affect nearly 40 % of children under the age of five (Black et al., 2013). Orange-fleshed sweet potato is one of the major biofortified crops and the most successful one in Africa. However, its susceptibility to major diseases remains a major hurdle in its production.

Viral pathogens present the greatest risk to sweet potato production, with 20–40 % of annual loss being attributed to viral infection alone (Clark and Hoy, 2006). The most detrimental disease caused by viruses, sweet potato virus disease (SPVD), is the result of coinfection of plants by positive-sense single-stranded RNA [(+)ssRNA] viruses: sweet potato feathery mottle virus (SPFMV), a potyvirus, transmitted by aphids, and sweet potato chlorotic stunt virus (SPCSV), a crinivirus, transmitted by whiteflies. The SPVD can cause yield losses of 80–90 % in infected plants (Karyeija et al., 1998; Kokkinos and Clark, 2006). Although SPCSV is known to have synergistic interactions and disease-inducing effects with other unrelated viruses (Untiveros et al., 2007; Karyeija et al., 2000), neither it, nor SPFMV alone consistently produce disease-related symptoms often contributing only to slight interveinal yellowing and circular spotting of leaves (Cuellar et al., 2009). However, coinfection with SPCSV and SPFMV can lead to a synergistic disease where SPFMV titers are capable of increasing several hundred- to thousand-fold in plants, leading to severe symptoms, including vein clearing, stunting, and yield loss that are associated with SPVD (Mukasa et al., 2006; Cuellar et al., 2008, 2009, Kokkinos and Clark, 2006a). The eukaryotic RNA silencing serves as a primary antiviral defense mechanism, via the production of virus-derived small-interfering RNAs (vsiRNAs) that interfere with the replicative ability of viral pathogens (Baulcombe, 2004). A Class I dsRNA specific RNase III enzyme (RNase3) encoded by the SPCSV genome has been shown to enhance the host RNA-silencing suppression activity of another SPCSV protein, p22 (Kreuze et al., 2005). Although p22 is capable of reducing host small interfering RNA (siRNA) accumulation on its own, RNase3 has not been found to significantly reduce host siRNA accumulation in the absence of p22 expression (Kreuze et al., 2005). Nevertheless, most SPCSV isolates lack the p22 gene and can still cause synergistic disease (Cuellar et al., 2008), and RNase3 by itself has been shown to be sufficient to provoke disease synergism in sweet potato plants (Cuellar et al., 2009).

MicroRNAs (miRNAs) are a class of small RNAs (sRNAs), typically 21–24 nucleotides (nt) in length, that can regulate host stress response and development (Han, 2019). Several miRNA families have been reported to play a direct role in plant defense response, via targeting and regulation of nucleotide-binding site leucine-rich repeat (NBS-LRR) disease resistance genes. Another role miRNAs play in defense response is in the biogenesis of phased secondary small interfering RNAs (phasiRNAs). PhasiRNA biogenesis is the result of miRNA-directed cleavage of certain messenger RNA (mRNA) transcripts, followed by RNA-dependent polymerization of the cleaved fragments, and subsequent processing by the DCL4 protein into 21-nt sRNAs, each of which is in phase with the miRNA cleavage site (Xia et al., 2015). Three major gene families encoding NBS-LRR proteins, pentatricopeptide repeat proteins (PPR), and MYB transcription factors, contribute to plant phasiRNA expression. phasiRNAs are associated with both plant defense and symbiosis, and studies have shown that miRNAs responsible for NBS-LRR-derived phasiRNA biogenesis are also responsible for DCL mRNA cleavage, indicating that phasiRNA biogenesis is closely coupled to the tight regulation of gene silencing and defense response (Zhai et al., 2011).

Global gene expression profiles of plants during viral pathogen infection can provide significant insights into plant-virus interaction dynamics and aspects of the host genome that are implicated in antiviral defense response. However, in sweet potato, very little is known about how viral infection affects host gene regulation at a systemic level. In this study, we generated transcriptome profiles of the SPVD-susceptible cultivar ‘Beauregard’, infected with SPCSV, SPFMV, or both, through deep sequencing of both mRNA and sRNA populations. Through comprehensive and integrated analyses of mRNA and sRNA profiles, we identified general biological features that defined sweet potato antiviral responses, as well as features that were unique to both duration and severity of viral infection. We were able to identify several essential pathways that were affected in sweet potato response by viral infection, largely related to biotic stress responses mediated by the hormone salicylic acid.

## 2 Materials and methods

### 2.1 Sample preparation and collection

Plants from the ‘Beauregard’ cultivar were inoculated with respective viruses via grafting on virus-infected *I. setosa* rootstocks on April 14, 2016. *I. setosa* rootstocks were either inoculated with SPFMV Piu3 isolate (GenBank ID: FJ155666), SPCSV m2−47 isolate (GenBank ID: HQ291259 for RNA1 and HQ291260 for RNA2), or a combination of the two. Additionally, ‘Beauregard’ plants grafted on healthy, uninfected *I. setosa* rootstocks were used as the mock group. Plants were kept in greenhouse conditions, and leaf tissues (the 3rd, 4th and 5th leaves from the top full-expanded leaf of each plant) were sampled at 3, 6 and 12 weeks after the initial grafting. The plants were pruned at the sixth week after graft-inoculation and allowed to regrow. Each experimental group contained three biological replicates, for a total of 36 samples (4 treatments × 3 time points × 3 biological replicates) (**Fig. S1**).

### 2.2 Library preparation and sequencing

RNA-Seq libraries were prepared according to the protocol described in Zhong et al. (2011). Briefly, mRNA was enriched by magnetic beads with Oligo (dT), and then sheered into short fragments in the fragmentation buffer. First strand cDNA was synthesized using SuperScriptIII reverse transcriptase (Invitrogen) in the presence of dNTPs. Second strand cDNA was synthesized using RNase H (NEB) and the Klenow fragment of DNA polymerase I (NEB) with a dUTP mix at 16 °C for 2.5 h using dUTPs. After end repair, adapters were ligated to the double stranded cDNA, and the dUTP-containing strand was degraded using a uracil DNA glycosylase. sRNA libraries were constructed following the protocol of Chen et al. (2012). Briefly, 10 ug of sRNA was fixed with 3’ and 5’ adapters via ligation. sRNAs were then reverse transcribed using SuperScript III reverse transcriptase, PCR enriched, separated on a 2% agarose gel, and gel purified to enrich fragments of the correct length. Both RNA-Seq and sRNA libraries were sequenced on an Illumina HiSeq 2500 system. Raw RNA-Seq and sRNA reads have been deposited in NCBI SRA under the accession number PRJNA649319.

### 2.3 RNA-Seq data processing and differential expression analysis

Raw RNA-Seq reads were processed using Trimmomatic (Bolger et al., 2014) to remove adapters and low-quality sequences, and trimmed reads shorter than 36 bases were discarded. The remaining reads were aligned to the SILVA rRNA sequence database (Quast et al., 2013) using Bowtie2 (Langmead and Salzberg, 2012) allowing up to 3 mismatches. Reads mapped to rRNA sequences were discarded. The final high-quality reads were aligned to the *Ipomoea trifida* reference genome (Wu et al., 2018) using HISAT2 (Kim et al., 2019) allowing up to 5 mismatches. Raw counts for each *I. trifida* gene model were generated by counting the total number of reads that were mapped to the gene region and normalized to reads per kilobase exon model per million mapped reads (RPKM). Differential expression analysis between control and infected samples was performed using DESeq2 (Love et al., 2014). Raw p values were adjusted for multiple testing using false discovery rate (FDR; Benjamini and Hochberg, 1995). Genes with a fold change ≥2 and an FDR < 0.05 were considered significantly differentially expressed. Gene ontology (GO) term enrichment analysis of differentially expressed genes was performed using the R package topGO (https://bioconductor.org/packages/topGO/). Geneious RNA (implemented in Geneious 11.1.5) was used to map RNA-Seq reads against genomes of SPCSV isolate M2−47 and SPFMV isolate Piu3 allowing up to 3 mismatches.

### 2.4 sRNA data processing and differential expression analysis

Raw sRNA reads were processed to remove adapters and trailing bases using a script provided in the VirusDetect package (Zheng et al., 2017). The remaining reads were further aligned to SILVA rRNA sequence databases (Quast et al., 2013) using Bowtie2 (Langmead and Salzberg, 2012) allowing up to 1 mismatch. Reads mapped to rRNA sequences were discarded. The remaining high-quality reads for each library were aligned to the *I. trifida* genome using ShortStack with default parameters (Axtell, 2013). miRNAs were identified using ShortStack for both individual libraries and a merged dataset incorporating sRNA reads from all libraries. PhasiRNAs were identified using PhaseTank with default parameters (Guo et al., 2014). Raw count data for unique sRNAs was generated using alignment files generated by ShortStack, and normalized to transcripts per million (TPM). To establish all miRNAs in our datasets, we included 21−22-nt sRNA sequences considered miRNA species by ShortStack in at least 2 of the 36 distinct samples, and 23−24-nt sRNAs in at least 4 (approximately 10 %) of our samples. The identified miRNAs were classified into different families based on the alignments of their sequences to known miRNAs in miRBase (Kozomara et al., 2018). Only unique sRNAs with a normalized TPM of 50 or greater in at least one library were included in differential expression analysis using DESeq2. sRNAs with a fold change ≥2 and an FDR < 0.05 were considered significantly differentially expressed. mRNA target prediction of miRNAs or phasiRNAs which exhibited significant differential expression was performed using Targetfinder (https://github.com/carringtonlab/TargetFinder). GO term enrichment analysis of miRNA and phasiRNA targets was performed using the R package topGO (https://bioconductor.org/packages/topGO/). Geneious RNA was used to map sRNA reads against genomes of SPCSV isolate m2−47 and SPFMV isolate Piu3 allowing one mismatch.

## 3 Results

### 3.1 Symptoms of sweet potato plants infected by SPCSV and/or SPFMV

**Fig. 1:**
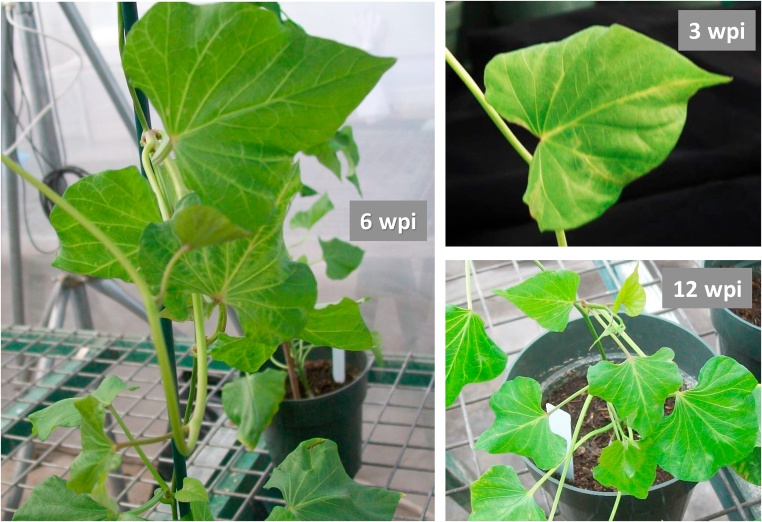
**Symptoms observed on leaves of ‘Beauregard’ plants coinfected with SPCSV and SPFMV at different time points.** wpi, weeks post inoculation. 3 wpi: vein clearing; 6 wpi: vein clearing, mosaic, and roll down; 12 wpi (plant pruned after 6 wpi): vein clearing and roll down.

We inoculated sweet potato plants with either SPCSV, SPFMV or both that caused SPVD. Symptoms induced by viruses were observed on inoculated sweet potato plants at three different time points [3-, 6- and 12-weeks post inoculation (wpi)]. In plants coinfected with SPCSV and SPFMV, intensity of symptoms increased from 3 to 6 wpi and then decreased at 12 wpi. Vein clearing was observed on leaves at 3 wpi, vein clearing, mosaic and roll down at 6 wpi, but only vein clearing and roll down at 12 wpi (Fig. 1). In SPCSV- or SPFMV-infected plants, very mild symptoms of roll down were observed at 6 and 12 wpi, while no clear symptoms were observed at 3 wpi (data not shown).

### 3.2 Transcriptome sequencing of sweet potato upon viral infection

Leaf samples were collected from virus-infected and mock plants, and used to construct and sequence both RNA-Seq and sRNA libraries (**Fig. S1**). A total of approximately 504 million RNA-Seq reads were generated across all 36 libraries, with an average of ∼14 million reads per library. After filtering out low-quality reads and reads corresponding to rRNA sequences found in the SILVA database (Quast et al., 2013), we obtained an average of ∼12.3 million cleaned reads per library. These reads were then mapped to the genome of *I. trifida* (Wu et al., 2018), a diploid wild relative of the hexaploid sweet potato, yielding an average mapping rate of approximately 75.0 % (**Table S1**). For sRNA libraries, we obtained a total of approximately 372 million raw reads, with an average of ∼10.3 million reads per library. After processing, a total of ∼129 million final cleaned reads and an average of 3.58 million reads per library were obtained. These cleaned reads were aligned to the *I. trifida* genome, resulting in a mapping ratio of ∼61.3 % (**Table S2**). Changes could be observed in different-sized sRNA species between different infections. Notably, ratios of 21-nt to 24-nt sRNA abundances, and 21-nt plus 22-nt to 24-nt sRNA abundances were significantly higher (p<5 × 10^−3^ and p<8 × 10^-4^, respectively) in coinfected plants (0.35 and 0.41, respectively) compared to other infections (0.15−0.16 and 0.17−0.2, respectively). The ratio of 21- to 22-nt sRNA abundances was also affected in coinfected plants (1.21) as compared to mock (1.62) or SPCSV (1.60) inoculated plants (p = 0.0008 and 0.0003 respectively), but not in SPFMV-inoculated plants (1.34).

Pearson correlation coefficients of RNA-Seq transcriptome profiles among different biological replicates were generally high, indicating the high reproducibility of our RNA-Seq data. This was further supported by the principal component analysis (PCA) of RNA-Seq transcriptome profiles (**Fig. S2**).

### 3.3 Viral transcripts and vsiRNAs in infected plants

**Fig. 2:**
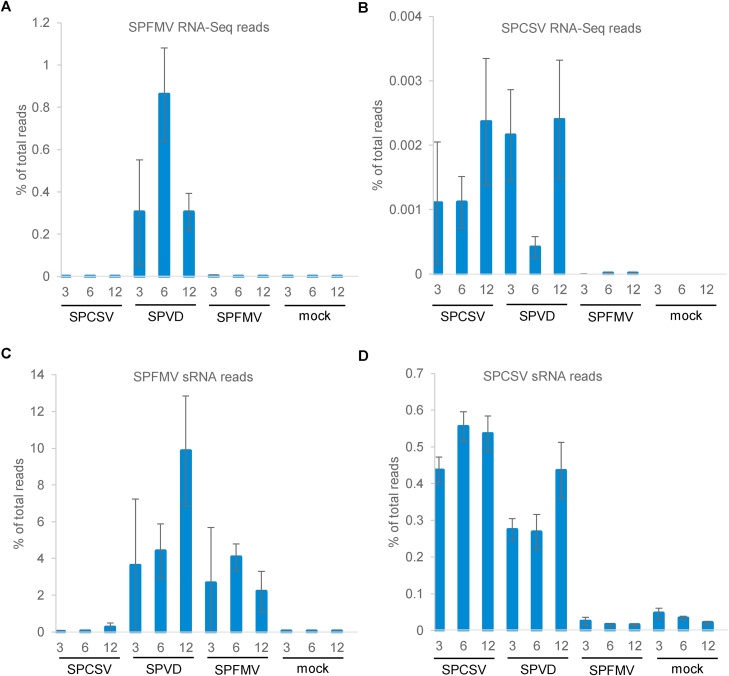
**Abundances of viral RNA transcripts and vsiRNAs in SPFMV and/or SPCSV-inoculated and mock-inoculated ‘Beauregard’ plants.** (**A-D**) Percentage of RNA-Seq **(A, B**) or vsiRNA reads (**C, D**) mapped to the SPFMV (**A, C**) or SPCSV (**B, D**) genomes at 3-, 6- and 12-weeks post inoculation. Data shown are mean and standard error of three biological replicates.

We also mapped RNA-Seq reads to the corresponding virus genomes, which resulted in extremely low mapping ratios (0.0005–1 %) in infected plants. The abundances of SPFMV-derived reads were extremely low in single infection, ranging from 0.0004 % at 12 wpi to 0.003 % at 3 wpi, and the former was in the range of background levels. Similarly, only 0.001−0.002% of reads were mapped to the SPCSV genome in single infection. In coinfected plants, reads mapped to the SPFMV genome were increased 338, 2779 and 806-fold compared to single infection at 3, 6 and 12 wpi, respectively, whereas SPCSV-derived reads were in a similar range of ∼0.002 % as compared to single infection with the exception of 6 wpi, at which SPCSV-derived reads were reduced to 0.0004 % (Fig. 2**A and B**). In interpreting these results, it should however be noted that SPCSV does not contain a poly A tail and the amounts of identified reads were therefore likely highly underrepresented compared to SPFMV and host transcripts.

vsiRNAs were more abundant than mRNAs for both viruses in all cases, but overall about 10-fold higher for SPFMV than SPCSV. The abundances of SPFMV vsiRNAs ranged from 2.2 to 9.8 % per sample while those of SPCSV vsiRNAs ranged from 0.24−0.55 %. SPFMV vsiRNA abundances were similar in singly or dually infected plants except at 12 wpi when they were about twice as high in coinfected plants. The abundances of SPCSV vsiRNAs in coinfected plants were about half of those in singly infected plants at 3 and 6 wpi, whereas they were only slightly less at 12 wpi (Fig. 2**C and D**).

A notable change could be detected in the abundance of different sizes of SPFMV vsiRNAs in different infections. In single infection, SPFMV vsiRNAs were predominantly of 22 nt with an average ratio of 21- to 22-nt vsiRNAs of 0.48, whereas this ratio was significantly different (p = 1.7 × 10^−7^) in coinfected plants (an average of 1.15). On the other hand, ratios of 21- to 22-nt SPCSV vsiRNAs were not significantly different in dually and singly infected plants, with an average ratio of 1.19 and 1.08, respectively.

### 3.4 Genes responsive to viral infections in sweet potato

**Fig. 3:**
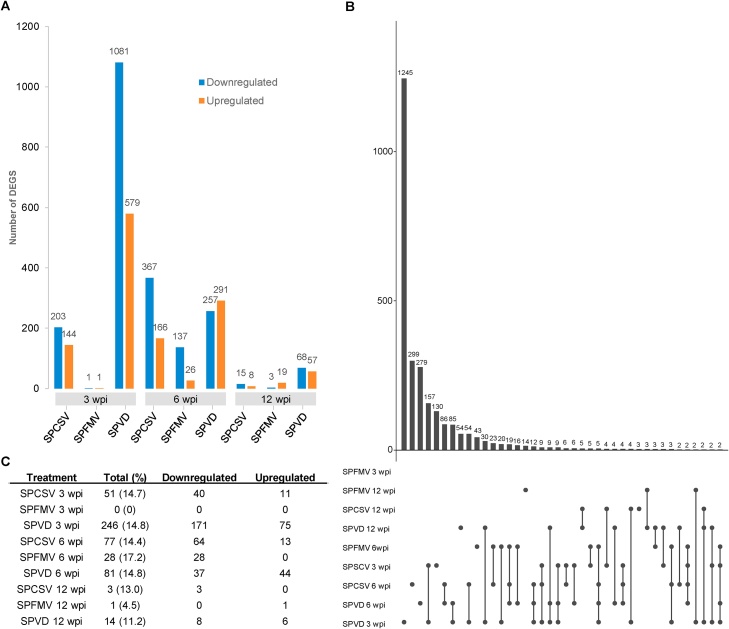
**Differential gene expression in response to SPCSV, SPFMV and SPVD in ‘Beauregard’. (A)** Number of differentially expressed genes (DEGs) at different time points after viral infection. **(B)** Intersection of DEGs between different viral infections. Vertical histogram bars indicate the number of DEGs and dots indicate the corresponding infections. **(C**) Number of frequency of DEGs annotated to be related to biotic stress response. wpi, weeks post inoculation.

We observed that the degree of differential gene expression was largely dependent on different viral infections. While SPVD-plants (coinfection of SPCSV and SPFMV) had much higher degrees of differential expression at 3 wpi [1,660 differentially expressed genes (DEGs)] compared to 6 and 12 wpi (548 and 125, respectively), both SPCSV and SPFMV-infected plants had overall higher degrees of differential expression at 6 wpi (533 and 163 DEGs, respectively) compared to 3 and 12 wpi (Fig. 3A). Differential gene expression in SPCSV-infected plants increased from 3 to 6 wpi then fell considerably at 12 wpi. Similarly, SPFMV infections substantially induced differential gene expression at 6 wpi compared to 3 wpi and 12 wpi (Fig. 3A). There were no genes that were differentially expressed across all infections and time points, and many genes were differentially expressed in only a single infection (Fig. 3**B**)

To better understand the effect that the three different virus infections had on sweet potato defense responses, DEGs annotated as related to responses to biotic stress were extracted. We found that an average of ∼14.6 % of all DEGs were biotic stress response-related. Overall, the largest number of biotic stress response-related genes were identified in coinfected plants at 3 wpi (246; Fig. 3C). Differentially expressed genes encoding WRKY transcription factors were enriched in coinfected plants at 3, 6 and 12 wpi, SPCSV-infected plants at 12 wpi and SPFMV-infected plants at 6 wpi. Additionally, genes encoding elicitor-activated 3−2 proteins were strongly downregulated by coinfection and SPCSV at 3 wpi. Genes encoding glycosyl hydrolase superfamily proteins were enriched in coinfected plants at 3 wpi (Table 1). Alcohol dehydrogenase was found to be downregulated by coinfection at 6 wpi, while genes encoding serine protease inhibitors were enriched in SPCSV infections at 3 and 6 wpi. Likewise, genes encoding protein kinase superfamily proteins were generally upregulated by the coinfection at all three time points (Table 1). In other infections, we found evidence for the downregulation of genes encoding serine hydrolase domain proteins by SPCSV at 3 and 6 wpi, and ethylene-forming enzymes by SPCSV and SPFMV at 6 wpi (Table 1). We also found evidence for the enrichment of downregulated genes encoding jasmonate-zim-domain (JAZ) proteins by SPFMV at 6 wpi. Interestingly, we found differentially expressed genes encoding leucine-rich repeat (LRR) family proteins were enriched in coinfected plants at 6 wpi (Table 1).

**Table 1:** **Enrichment of response to biotic stress-related DEGs in ‘Beauregard’ upon viral infection.** DEGs with GO terms related to response to biotic stress were functionally annotated, and the annotations with the highest frequencies in each infection are shown.

Infection	Function	Downregulated	Upregulated
**SPCSV 3 wpi**	elicitor-activated gene 3−2	4	0
	serine hydrolase (FSH1) domain containing protein	4	0
	integrase-type DNA-binding superfamily protein	3	0
	heat shock protein	2	0
	tubby like protein	0	2
**SPFMV 3 wpi**	–	–	–
**SPVD 3 wpi**	WRKY DNA-binding protein	5	5
	elicitor-activated gene 3−2	10	0
	glycosyl hydrolase superfamily protein	2	6
	leucine-rich receptor-like protein kinase family protein	3	5
	conserved hypothetical protein	2	3
**SPCSV 6 wpi**	conserved hypothetical protein	5	0
	ethylene-forming enzyme	4	0
	serine protease inhibitor, potato inhibitor I-type family protein	0	3
	NAC (No Apical Meristem) domain transcriptional regulator	3	0
	AAA-ATPase	3	0
**SPFMV 6 wpi**	jasmonate-zim-domain protein	4	0
	ethylene-forming enzyme	3	0
	basic helix-loop-helix (bHLH) DNA-binding family protein	3	0
	WRKY DNA-binding protein	1	0
**SPVD 6 wpi**	alcohol dehydrogenase	4	0
	WRKY DNA-binding protein	2	1
	conserved hypothetical protein	2	1
	leucine-rich repeat (LRR) family protein	3	0
	protein kinase superfamily protein	0	3
**SPCSV 12 wpi**	glucose-6-phosphate/phosphate translocator	1	0
	WRKY DNA-binding protein	1	0
	plant invertase/pectin methylesterase inhibitor superfamily	1	0
**SPFMV 12 wpi**	protein phosphatase 2C family protein	0	1
**SPVD 12 wpi**	WRKY DNA-binding protein	0	2
	protein kinase superfamily protein	0	1
	TIFY domain/Divergent CCT motif family protein	1	0
	pathogenesis-related	0	1
	elicitor-activated gene 3−2	1	0

**Fig. 4:**
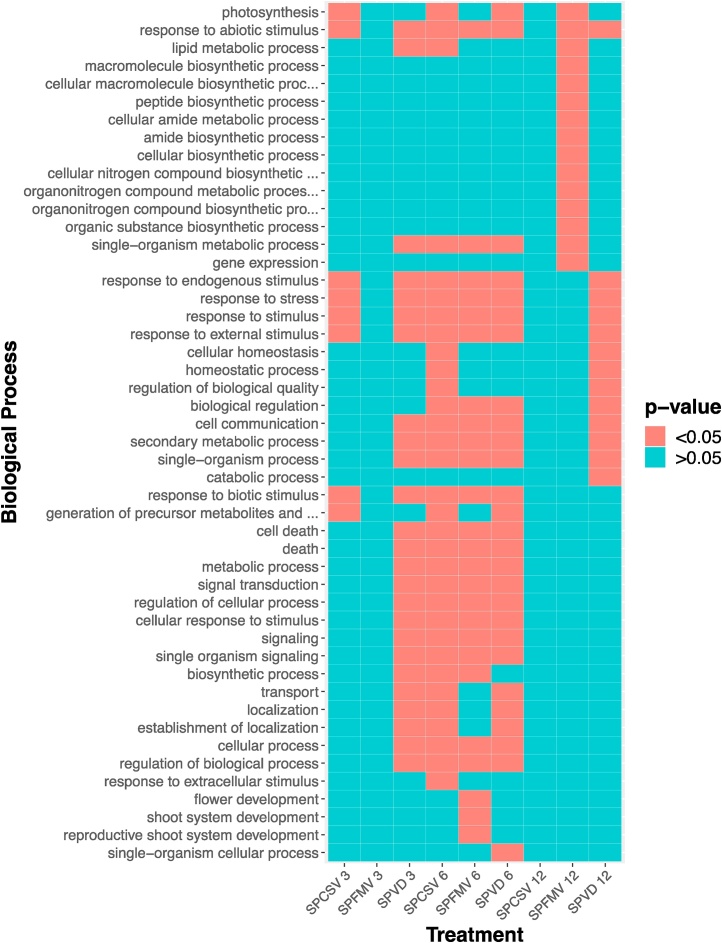
**GO terms enriched in genes responsive to viral** i**nfections in ‘Beauregard’.** Acronyms shown along the x-axis are relate to disease infection groups, while numbers indicate the weeks post inoculation.

GO term analysis of DEGs revealed that processes involved in response to general stressors were significantly enriched in multiple different infections, and several processes involved in response to biotic stimulus, secondary metabolism, and cell death were highly enriched in many if not all of the infections. Interestingly, we found that biological processes related to signaling and stress response were mainly significantly enriched in infections at 6 wpi as opposed to infections at 3 or 12 wpi, while those related to metabolism were significantly enriched at 12 wpi, largely in the SPFMV infection. Viral infection appeared to have a general, rather than a virus-specific effect on plant response (Fig. 4).

### 3.5 miRNAs and phasiRNAs responsive to viral infections in sweet potato

**Fig. 5:**
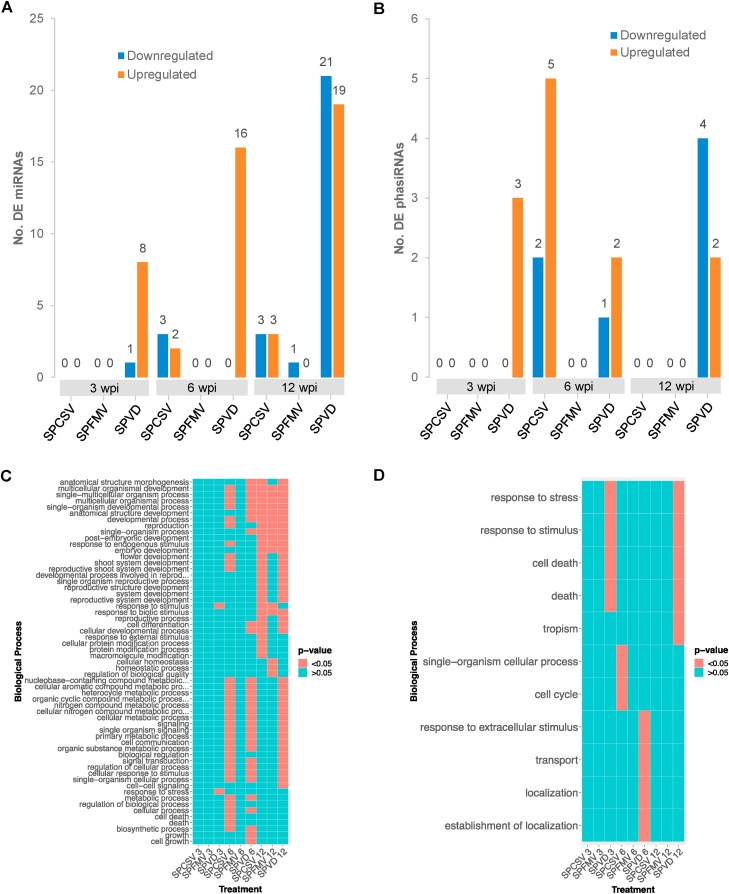
**Differential expression of sRNAs in ‘Beauregard’ upon viral infection. (A,B)** Number of differentially expressed (DE) miRNAs **(A)** and phasiRNAs **(B)** upon viral infection. **(C,D)** GO terms significantly enriched in the putative targets of differentially expressed miRNAs **(C)** and phasiRNAs **(D)**. Acronyms shown along the x-axis are relate to disease infection groups, while numbers indicate the weeks post inoculation (wpi).

Differential expression of miRNAs was strongly correlated to viral infection as well as sampling time point. SPCSV infections had very few differentially expressed (DE) miRNAs until 12 wpi and SPFMV infections had little to no DE miRNAs at any time points, whereas coinfections showed overall higher degrees of miRNA differential expression at all sampling time points (Fig. 5A). DE miRNAs were found to belong to a diverse number of families, including 14 previously characterized and 25 novel families (**Table S3**). Among the novel DE miRNAs, two were predicted to target NB-ARC (nucleotide-binding adaptor shared by APAF-1, R proteins, and CED-4) domain-containing disease resistance genes, one of which was upregulated by coinfection at 3 wpi while downregulated at 12 wpi. Another downregulated miRNA belonging to the miR396 family was also predicted to target NBS-LRR disease resistance genes. Two unique miRNAs belonging to the miR390 family predicted to target LRR receptor-like protein kinase genes were found to be differentially expressed in the coinfection at 6 or 12 wpi, one of which was differentially expressed in both time points as well as in the SPCSV infection at 12 wpi. Furthermore, we identified three unique DE miRNAs predicted to target Argonaute family genes, of which two belonged to the family miR396 and one belonged to the miR403 family. One of the miRNAs belonging to the miR396 family was downregulated by all three infections at 12 wpi. The other two DE miRNAs predicted to target Argonaute family genes were downregulated by the coinfection at 12 wpi.

We identified a similar distribution of DE phasiRNAs, the majority of which were either differentially expressed in samples with coinfection or samples inoculated with SPCSV at 6 wpi (Fig. 5B). Of these DE phasiRNAs, four were predicted to target NB-ARC and NBS-LRR disease resistance genes, with one downregulated by coinfection at 12 wpi, one upregulated by SPCSV at 6 wpi, and two downregulated by coinfection at 12 wpi while also upregulated by coinfection at 3 wpi. We also identified two DE phasiRNAs that were predicted to target receptor-like protein kinase (RLK) mRNAs, one of which was downregulated by SPCSV at 6 wpi, but upregulated at both 3 and 6 wpi (**Table S4**).

### 3.6 Correlation between mRNA and sRNA differential expression profiles

In large part we saw very little correlation between expression of DE miRNAs/phasiRNAs and their respective predicted target mRNAs. We did identify one gene encoding a bHLH transcription factor that was significantly downregulated while a miRNA belonging to the miR159 family predicted to target this gene was significantly upregulated by SPCSV at 6 wpi (**Fig. S3**). The fact that we did not see much evidence supporting the expected negative correlation between miRNA profiles and corresponding target mRNA profiles, suggests that antiviral defense may be regulated by miRNAs at the translational level, rather than at the post-transcriptional gene silencing level.

Based on our phasiRNA differential expression analysis, we found evidence of two genes that were significantly downregulated in the presence of significantly upregulated phasiRNAs predicted to target said transcripts. One of these was a gene encoding a putative disease resistance (TIR class) protein downregulated by SPCSV at 6 wpi, while the other was a gene encoding a phosphofructokinase family protein downregulated by the coinfection also at 6 wpi.

GO analysis of the target genes of DE miRNAs and phasiRNAs on the other hand indicated that many biological processes and pathways that were enriched in our differential gene expression analysis were also targeted by DE sRNAs. Pathways that were heavily targeted by DE miRNAs included response to external and biotic stimuli, cell death regulatory pathways, development, homeostasis, and signaling (Fig. 5C). DE phasiRNAs were found to target similar pathways, although more acutely focused on processes related to stress response and cell death (Fig. 5D).

## 4 Discussion

In the present study we set out to uncover biological pathways underlying the transcriptome responses of a susceptible orange-fleshed sweet potato cultivar, ‘Beauregard’, to SPCSV, SPFMV, and coinfection by both viruses that causes SPVD. A previous study also investigated the effect of these viruses on host sweet potato transcriptomes using a cDNA microarray containing 2,765 features (Kokkinos et al., 2006) however, our current understanding on genome-wide transcriptome responses of sweet potato to its two most impactful viruses, SPCSV and SPFMV, remains very limited.

Our finding that SPFMV derived transcripts were close to the background levels in singly infected plants is consistent with several previous reports in sweet potato (Cuellar et al., 2009; Karyeija et al., 2000; Kokkinos and Clark, 2006). Coinfection with SPCSV and SPFMV, however, led to several hundred-fold increase of SPFMV transcripts whereas SPCSV transcript levels were not affected much as compared to SPCSV single infection, which is also consistent with previous studies. In contrast to SPFMV transcripts, differences in SPFMV vsiRNAs were only detected at 12 wpi in coinfected plants, where they were more than two-fold higher than in other infections and timepoints (Fig. 2). The increase in SPFMV vsiRNAs at 12 wpi correlated with a reduced SPFMV transcript accumulation compared to 6 wpi indicated an increase in RNA silencing-mediated control of virus replication at later stages of the infection. The increased SPFMV vsiRNA levels found at 12 wpi is comparable, though far less extreme to what has been found in previous studies with cultivar ‘Huachano’ (Cuellar et al., 2015; Kreuze et al., 2009, 2020), and may thus be a general outcome of prolonged infection with SPFMV and SPCSV in susceptible cultivars. A significant change from 22- to 21-nt SPFMV-derived siRNAs observed in coinfected plants compared to singly infected plants might be related to failure of RNA silencing mediated control of SPFMV as it was correlated with increased SPFMV transcripts. As the ratios of 22- to 21-nt SPCSV vsiRNAs in single and dual infection were not significantly different from each other and similar to that of SPFMV in coinfected plants, one could hypothesize that SPCSV may interfere with the production of 22-nt vsiRNAs, leading to a relative increase in 21-nt vsiRNAs. As a consequence of increased SPFMV transcripts in coinfected plants, expression of SPFMV encoded HC-Pro could further affect sRNA sizes in plants leading to the overall increase in 21- and 22-nt sRNAs as compared to 24-nt sRNAs in coinfected plants. Indeed, a shift from 24- to 21−22-nt sRNAs appears to be a general consequence of several potyviral infections as reported previously for different plant/potyvirus combinations (Prigigallo et al., 2019; Tatineni et al., 2014).

Our analysis corroborated evidence of both specific and general aspects of sweet potato viral defense response (Whitham et al., 2003), as well as provided novel insights into regulatory networks of sweet potato-virus interactions. We found evidence for the enrichment of several functional gene classes in response to viral infection (Fig. 4**)**. Photosynthetic pathways in coinfected plants in the early to mid-infection stages were significantly affected, likely contributing to the mosaic and yellowing symptoms found in these plants (Fig. 1). Genes involved in cell signaling and communication were highly affected by viral infection between 3 and 6 wpi, consistent with studies describing RNA virus effect and reliance on host trafficking machinery (Hong and Ju, 2017; Whitham et al., 2003) as well as viral protein and host protein co-localization (Weinheimer et al., 2016). Similarly, cell death and defense pathways were significantly induced at 6 wpi, consistent with SPVD symptoms observed at the same time (Fig. 1). Induced alterations of primary and secondary metabolisms in response to viral infection were enriched in nearly all virus infections, consistent with findings described in other plant-virus systems (Fernández-Calvino et al., 2014; Whitham et al., 2003). Additionally, many pathways involved in general stress responses were enriched in the mid to late stages of infection in all three virus infections, suggesting that although symptoms of plants infected with SPFMV or SPCSV are often mild, these viruses still have an impact on host homeostasis.

Many biotic stress response-related gene families that are regulated by salicylic acid (SA), a hormone involved in endogenous cell signaling and plant defense against pathogens (War et al., 2011), were enriched across various viral infections and time points. These included the elicitor-activated gene 3−2 family, the ethylene-forming enzymatic family, the alcohol dehydrogenase, and the WRKY transcription factor family (Table 1). WRKY transcription factors bind to the *NPR1* promoter in Arabidopsis in an SA-dependent manner (Jayakannan et al., 2015), and are involved in the NPR1 activation and regulation of plant systemic acquired resistance (SAR) (Yu et al., 2001). Consistent with the approximately equal distribution of significantly up- and down-regulated WRKY transcription factor genes, we found an equal distribution of up- and down-regulated NPR-like genes across multiple infections at multiple time points (data not shown). Additionally, we found that several genes positively controlled by hormonal pathways involved in plant defense, including gibberellin and cytokinin pathways, were downregulated, although expression of genes regulated by these pathways was not nearly as enriched as genes regulated by the SA pathway, suggesting the importance of SA as a mediator of host response to viral infection.

Compared to mRNA differential expression patterns, DE sRNA patterns were generally more temporally and infection specific; the majority of DE miRNAs were observed in the coinfection at 12 wpi, while the majority of DE phasiRNAs were observed in SPCSV and coinfections at 6 and 12 wpi, respectively. While all but one DE miRNA that was predicted to target NB-ARC domain-containing disease resistance genes belonged to previously undescribed miRNA families, we did identify several known families represented by DE miRNAs that have been reported to play roles in both general and specific plant defense response pathways, including miR160 (Natarajan et al., 2018), miR396 (Chandran et al., 2019), miR171 (Tong et al., 2017), miR159 (Liang et al., 2019), miR168 (Várallyay et al., 2010), miR394 (Tian et al., 2018) and miR530 (Liang et al., 2019). Furthermore, consistent with our DE mRNA results, several DE miRNA families, including miR156, miR160 and miR390, have been shown to play a role in the SA pathway regulation (Yin et al., 2018; Natarajan et al., 2018; Marin et al., 2010). Our DE mRNA analysis suggested that viral pathogens may work to suppress SA-mediated defense response pathways, either through direct interference, or through promotion of pathways known to be antagonistic to SA-related pathways, like the auxin-mediated growth response pathway (Natarajan et al., 2018). Downregulation of miR160 has been linked to reduced SA-mediated defense response and lowered SAR (Natarajan et al., 2018). The fact that these families were downregulated in most instances further supports that viral infection targets SA pathways in sweet potato. Interestingly, overexpression of miR156 has been linked to SA suppression in plants (Yin et al., 2018), yet we consistently observed miR156 downregulation in all infections in which it was differentially expressed. This could suggest a basal defense response or an aspect of the SA-mediated defense response pathway that remains untargeted by viral pathogens.

Similar to miRNA findings, DE phasiRNAs were largely confined to the coinfections and the SPCSV infection at 6 wpi. Consistent with previous reports (Fei et al., 2013), many of the phasiRNA-generating loci (PHAS loci) that produced DE phasiRNAs in sweet potato responses to viral infections corresponded to NB-ARC and NBS-LRR genes. Individual phasiRNAs arising from these loci were largely found to be upregulated prior to 12 wpi while downregulated at 12 wpi. One possible explanation for this pattern is that the host is reliant on these siRNAs to counter potentially harmful levels of NBS-LRR accumulation during the early stages of infection. Interestingly, although we found evidence of several DE miRNAs targeting NBS-LRR genes, none of the PHAS loci which produced DE phasiRNAs in our analysis were predicted to be targeted by our DE miRNAs.

In conclusion, this study characterized transcriptome responses of sweet potato to single- or coinfection of the two most economically important viruses, SPCSV and SPFMV, at both mRNA and sRNA levels. Thousands of genes and dozens of miRNAs and phasiRNAs including several novel ones were identified to be responsive, mainly to the coinfection of the two viruses. Several SA-mediated pathways were found to play important roles in the antiviral defense response of sweet potato. These findings provide unique insight into the sweet potato-virus interaction and provide critical information for facilitating viral management.

## Author contributions

ZF and JK: Conceptualization, Funding acquisition, Methodology, Supervision, Validation, Writing - review & editing. Project administration. SF: Investigation, Methodology. MD: Investigation. RB and JK: Formal analysis, Visualization. RB: Writing - original draft, Writing - review & editing.

## Declaration of Competing Interest

The authors report no declarations of interest.
