# Supplementary material for: Transcriptome analysis provides insights into the responses of sweet potato to sweet potato virus disease (SPVD)
Source: Virus Res. 2021 Apr 2;295:198293. doi: 10.1016/j.virusres.2020.198293 (PMC7985617; doi:10.1016/j.virusres.2020.198293)
Supplement: Supplementary file 1 [file mmc1.pdf]

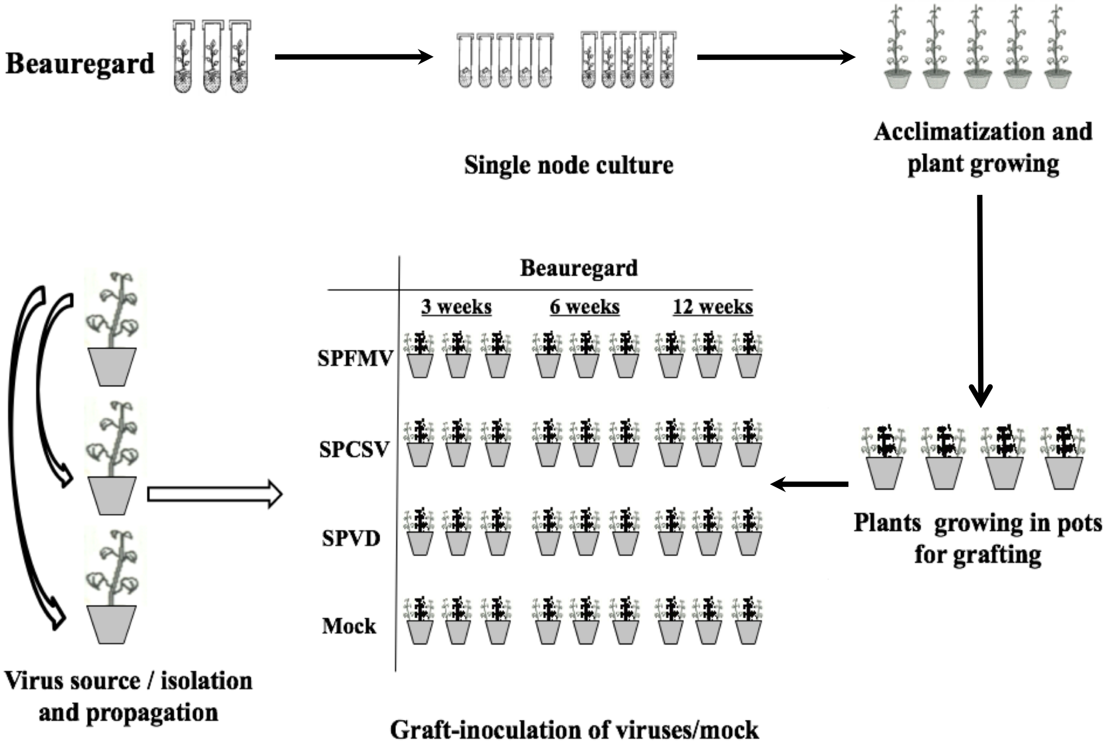

Figure S1. Schematic diagram of the overall experimental design

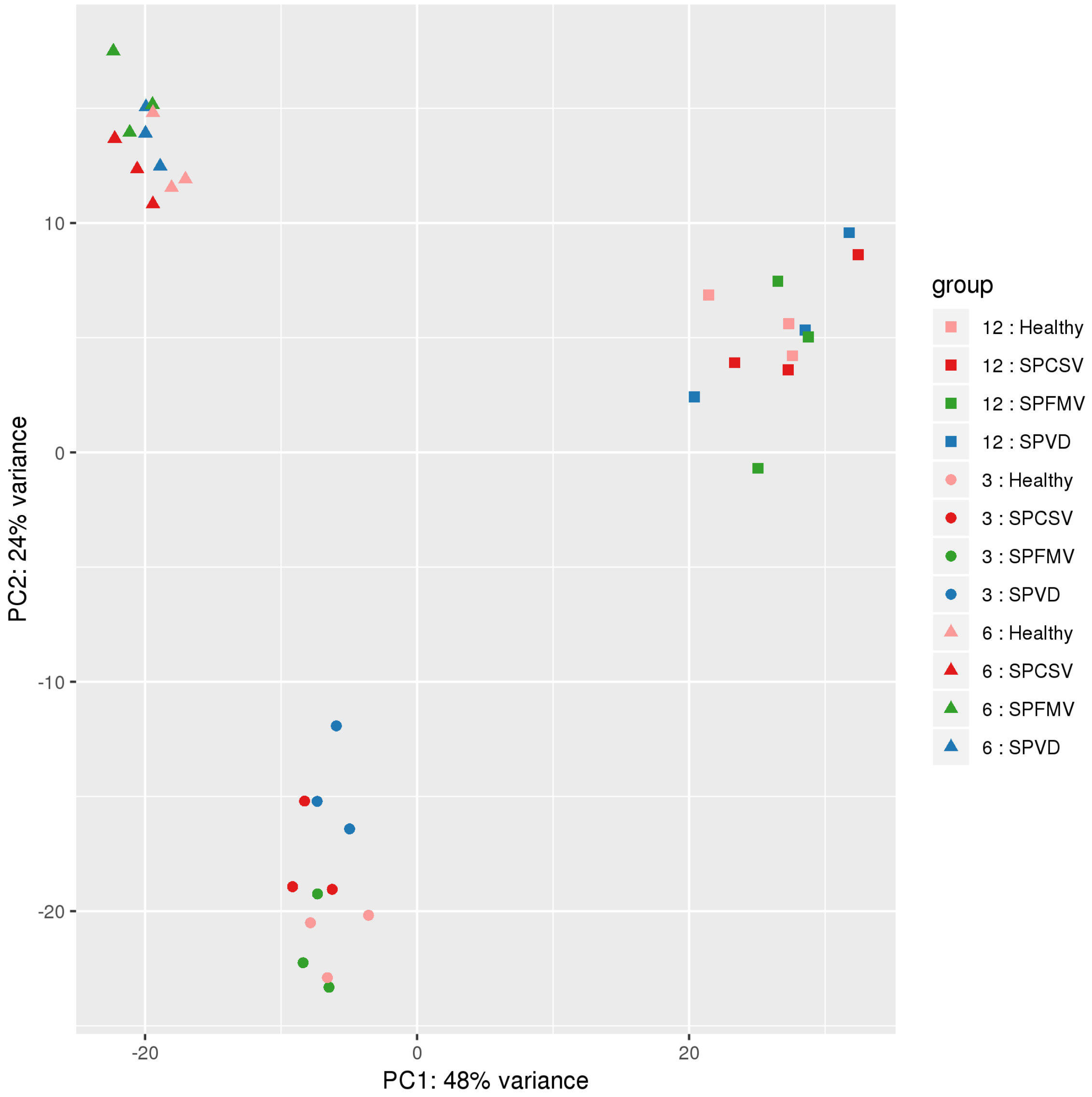

Figure S2. Principle component analysis (PCA) of RNA-Seq libraries

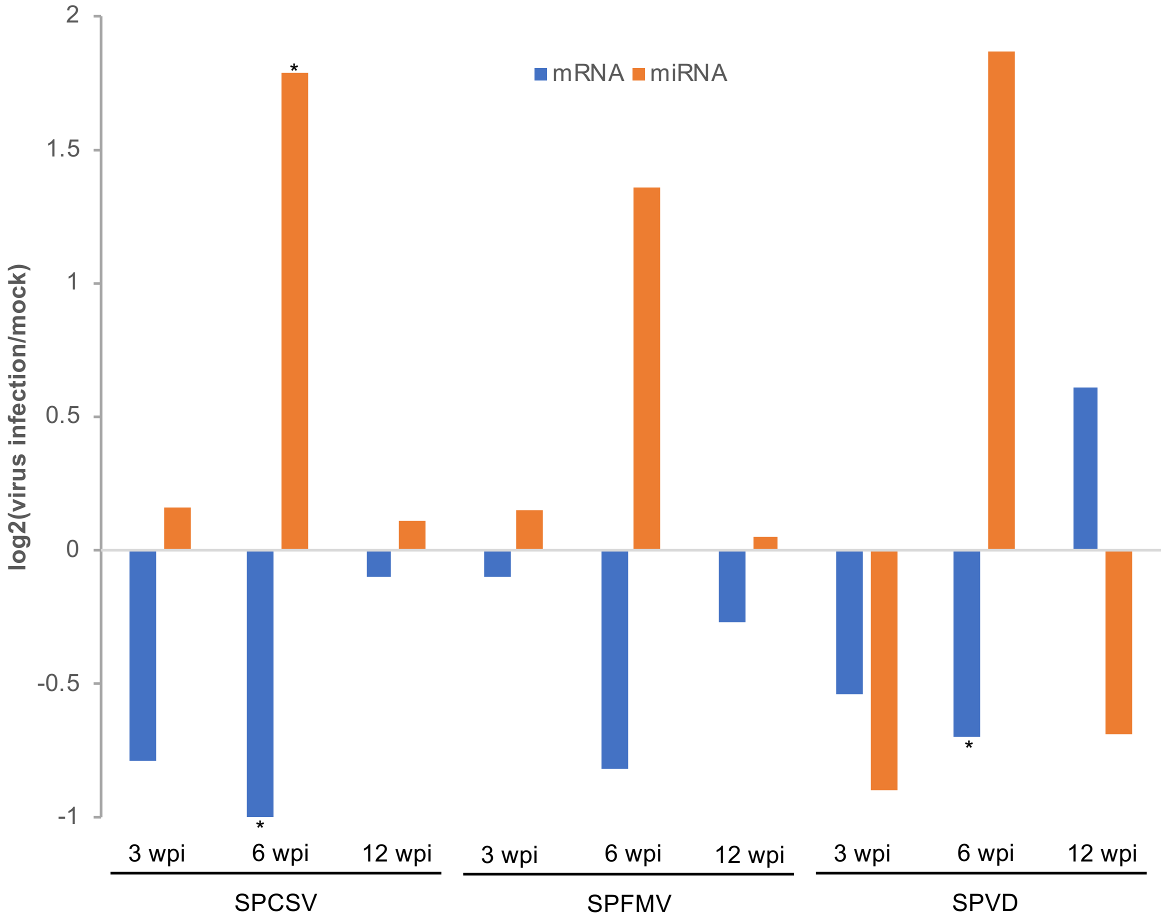

Figure S3. Expression dynamics of miRNA159 (UUUGGAUUGAAGGGAGCUCUG) and its potential target gene *itf12g00110* (encoding a bHLH transcription factor) upon viral infection. The bar chart shows log2 fold change in expression of miRNA159 and *itf12g00110* in SPCSV-infected, SPFMV-infected and coinfected (SPVD) plants compared to mock plants. * means the corresponding fold change is statistically significant (adjusted $p < 0.05$). wpi: weeks post inoculation.
